# Disseminated Tuberculosis in a Patient on Tumor Necrosis Factor (TNF)-α Inhibitor Treatment for Ankylosing Spondylitis: A Case Report

**DOI:** 10.7759/cureus.72796

**Published:** 2024-10-31

**Authors:** Lana Fadl, Arowa Abdelgadir, Hibah Mirza, Aqsa Mahreen, Siddalingana Gouda Thaplar G Gouda

**Affiliations:** 1 Internal Medicine, Walsall Healthcare NHS Trust, Walsall, GBR; 2 Internal Medicine, Royal Hampshire County Hospital, Winchester, GBR; 3 Microbiology, Walsall Healthcare NHS Trust, Walsall, GBR; 4 Hospital Medicine, Walsall Healthcare NHS Trust, Walsall, GBR; 5 Acute Medicine, Walsall Healthcare NHS Trust, Walsall, GBR

**Keywords:** adalimumab, ankylosing spondylitis, anti-tnf-α treatment, disseminated tuberculosis, miliary tuberculosis

## Abstract

Tumour necrosis factor-alpha (TNF-α) inhibitors are commonly used in the treatment of ankylosing spondylitis (AS) due to their effectiveness in reducing inflammation and slowing disease progression. However, their use is associated with an increased risk of opportunistic infections, particularly tuberculosis (TB). This case report presents a young male patient in the United Kingdom (UK) with AS, who had been on long-term biological therapy with adalimumab, a TNF-α inhibitor. The patient developed disseminated TB, which rapidly progressed and unfortunately resulted in the patient's death. This case underscores the importance of comprehensive screening for latent TB before initiating TNF-α inhibitor therapy, as well as ongoing monitoring throughout treatment. Given the multicultural nature of the UK, where individuals may be exposed to TB without traveling to endemic areas, careful attention to TB risk across all ethnicities is critical. This case highlights the need for heightened vigilance and tailored preventive strategies to mitigate the risks of TNF-α therapy.

## Introduction

Tumor necrosis factor (TNF) plays a pivotal role in the inflammatory cascades characteristic of ankylosing spondylitis (AS), particularly in activating immune cells and perpetuating chronic inflammation in the spinal column and sacroiliac joints. Elevated TNF levels are frequently observed in AS patients, contributing to disease progression and symptomatology. TNF-α inhibitors have demonstrated efficacy in managing AS by mitigating inflammation and enhancing functional mobility. Adalimumab, certolizumab pegol, etanercept, golimumab, and infliximab are recommended treatment options for severe active AS in adult patients unresponsive to or intolerant of non-steroidal anti-inflammatory drugs [1].

Of note, adalimumab, a monoclonal antibody targeting tumour TNF-alpha (TNF-α), has been associated with an elevated risk of tuberculosis (TB) compared to other anti-TNF-α agents. However, the magnitude of this risk varies across studies. Meta-analytical findings suggest that the overall risk of developing TB with anti-TNF-α therapy, including adalimumab, is not significantly different from that in untreated populations [2]. However, real-world data from Taiwan indicate a heightened incidence of TB events with adalimumab compared to etanercept. Notably, literature reviews highlight a three to four times greater TB risk with adalimumab and infliximab relative to etanercept [3].

While adalimumab poses a risk of TB, this risk appears to be lower than that associated with infliximab and higher than with etanercept [4]. Therefore, careful screening and continuous monitoring are imperative, particularly in high-risk patient cohorts. An unusual case is presented here involving a patient with AS who developed an aggressive form of disseminated TB following treatment with anti-TNF-α therapy, unfortunately resulting in a fatality, emphasizing the unforeseen severity and rapid progression to mortality observed in such clinical scenarios. Noteworthy is the scarcity of documented cases portraying swift deterioration to death due to disseminated TB in the context of anti-TNF-α therapy.

## Case presentation

A 40-year-old male of white British ethnicity with a history of psoriasis received a diagnosis of AS in his early 20s. He had been receiving nonsteroidal anti-inflammatory drugs (NSAIDs) for 13 years as treatment but with inadequate symptom control. In 2015, after a loss in follow-up, he was re-evaluated at the rheumatology clinic. With a Bath Ankylosing Spondylitis Disease Activity Index (BASDAI) score of 8, the decision was made to start anti-TNF-α therapy for better disease management. Prior to commencing anti-TNF-α treatment, screening tests, including hepatitis screening and chest radiography, were conducted to exclude TB. No abnormalities were identified in these investigations. The patient had no recent travel history and no previous contact with individuals diagnosed with TB. The patient was started on anti-TNF-α therapy, specifically golimumab, which led to a notable improvement in his symptoms, as evidenced by a reduction in the BASDAI score. After two years on golimumab, the patient developed side effects from the medication; hence, he was switched to adalimumab, with symptoms remaining relatively stable.

On January 18, 2024, the patient presented with respiratory distress, fever, night sweats, abdominal distension, and weight loss, with symptoms persisting for just over three weeks. Investigations revealed the results presented in Table 1.

**Table 1:** Laboratory results ((18/01/2024)

Test	Result	Reference Range	Abnormality
ALP	985	30-130 IU/L	Above Normal
ALT	78	0-41 IU/L	Above Normal
GGT	338	0-71 IU/L	Above Normal
CRP	256	0-5 mg/L	Above Normal
WCC	7.7	4-10 × 10⁹/L	Normal
Hb	115	130-180 g/L	Below Normal
PLT	210	150-450 × 10⁹/L	Normal
Neutrophils	6.1 (81%)	1.8-7.7 × 10⁹/L	Normal
Lymphocytes	1.1 (14%)	1-4.8 × 10⁹/L	Normal

**Figure 1:**
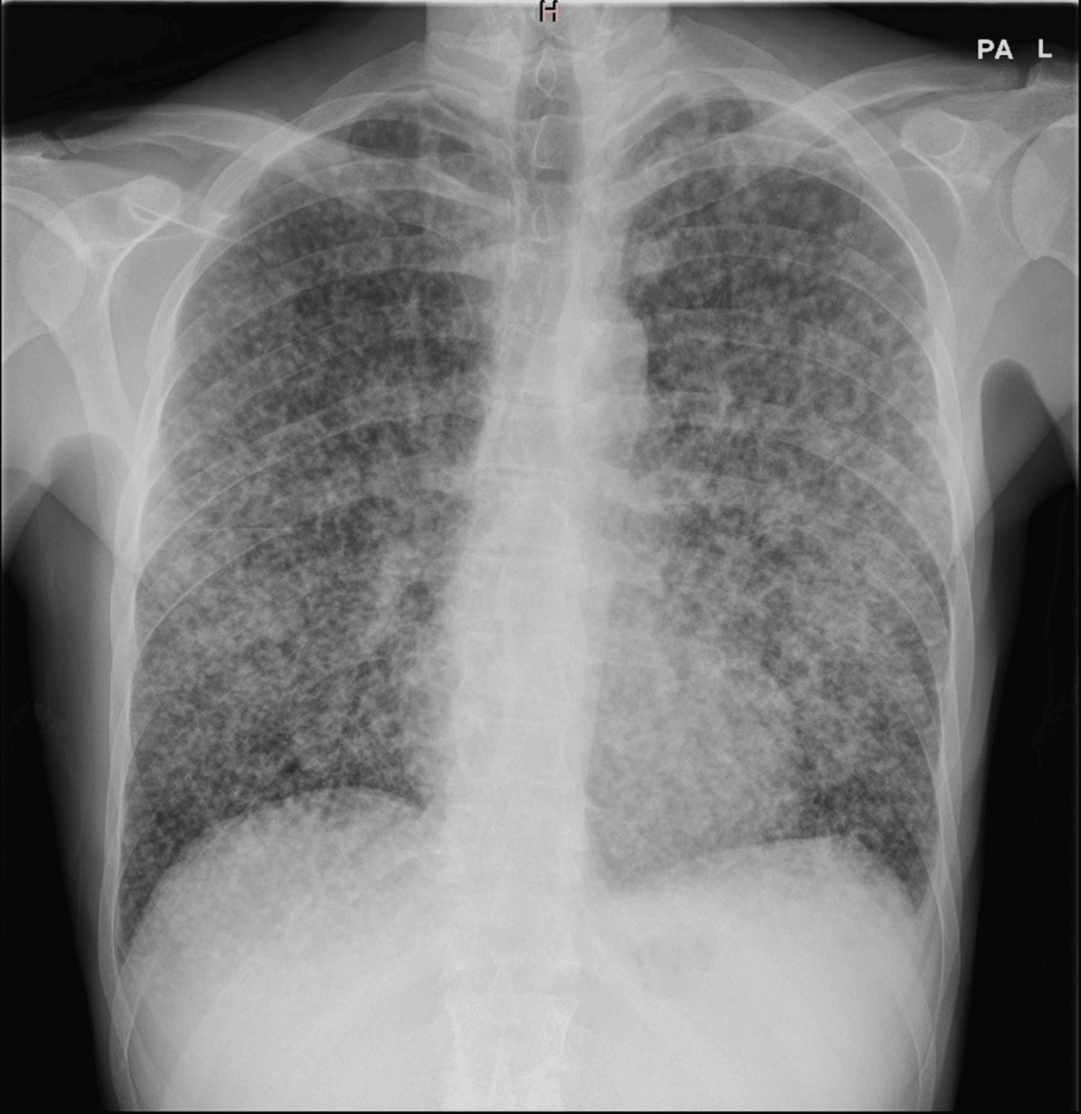
Chest X-ray of this patient showing disseminated tuberculosis

The chest X-ray (Figure 1) revealed multiple diffuse nodular opacities throughout both lungs, which suggested miliary TB. Sputum culture for mycobacterium TB was positive, which confirmed the diagnosis.

**Figure 2:**
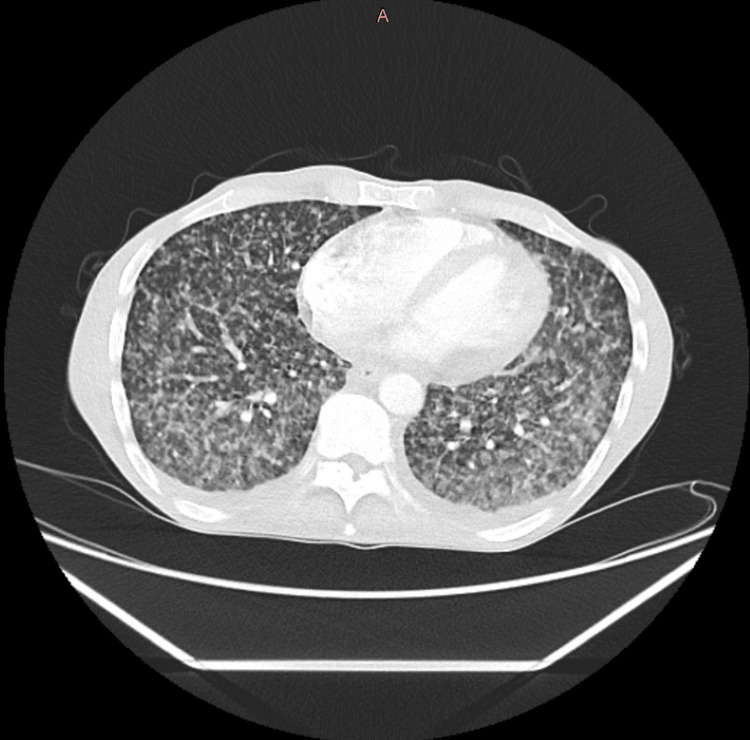
CT thorax with contrast of this patient showing disseminated tuberculosis

CT thorax-abdomen-pelvis (CT-TAP) with contrast (Figure 2) showed extensive nodularity throughout the lungs with individual nodules measuring between 2 and 3 mm nodularity extended along the fissures, and no effusion was detected. The liver appeared slightly enlarged. The spleen was enlarged at 16 cm and contained multiple low-density foci. There was a trace of free fluid. Bowel calibre was normal throughout. Chronic fusion of the sacroiliac joint was noted. Appearance was consistent with military TB.

Anti-tuberculous treatment was initiated on January 22, 2024, after consulting with rheumatology services, which advised discontinuing adalimumab pending further evaluation in four months. The patient was subsequently discharged under the care of the TB team for ongoing monitoring. One-week post-discharge, elevated liver function tests were noted, with alanine aminotransferase (ALT) at 235 IU/L and alkaline phosphatase (ALP) at 843 IU/L, resulting in the withholding of anti-TB medications in accordance with hospital protocols. Despite continued monitoring, liver enzyme levels remained significantly elevated, preventing the reintroduction of the anti-TB treatment.

**Figure 3:**
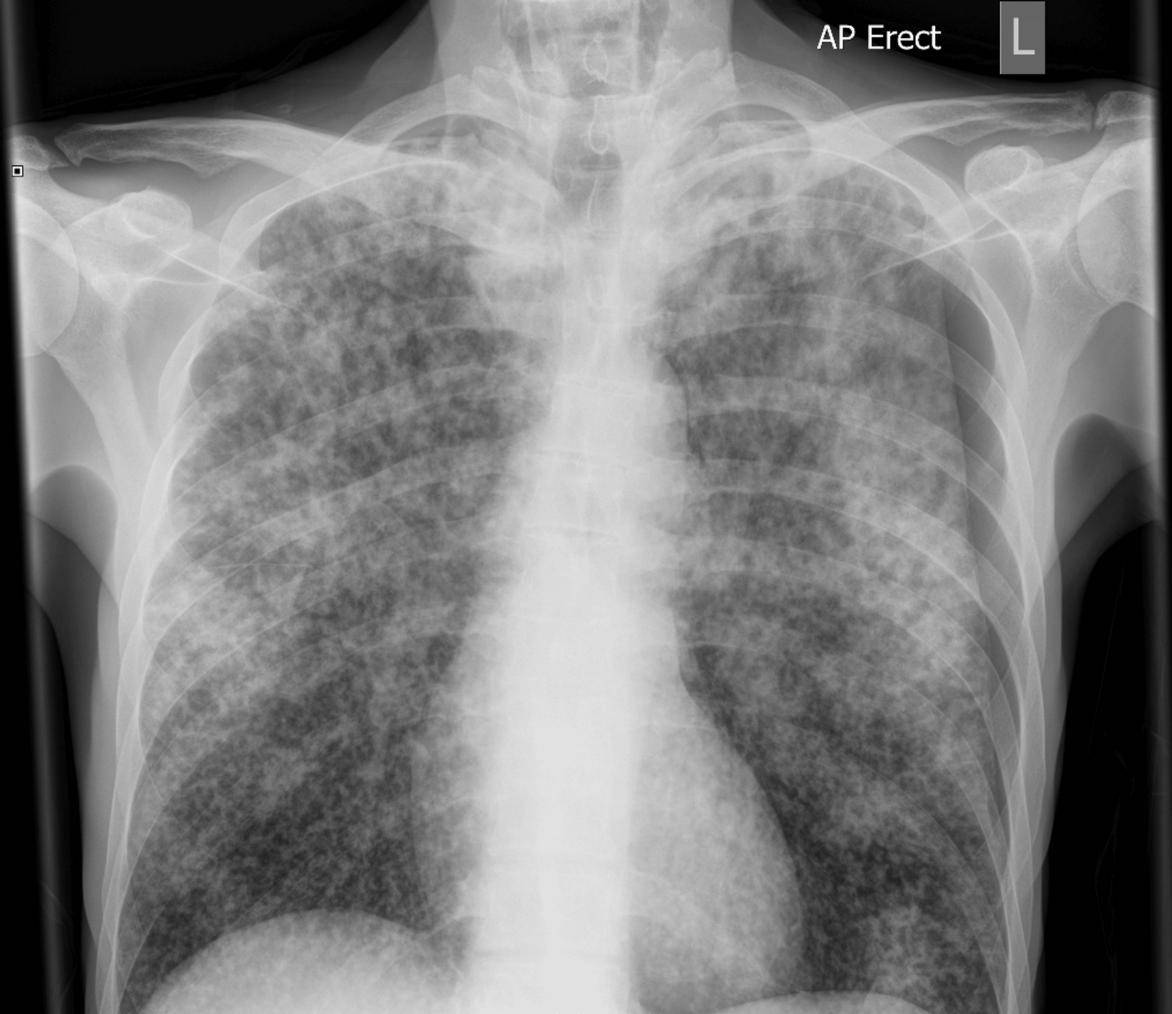
Repeat CXR (07/03/2024) showing extensive miliary TB and small pneumothorax

On March 7, 2024, the patient was readmitted due to worsening respiratory distress and weight loss. A repeat chest X-ray (Figure 3) showed extensive miliary changes throughout both lung fields, along with a small left-sided pneumothorax.

**Figure 4:**
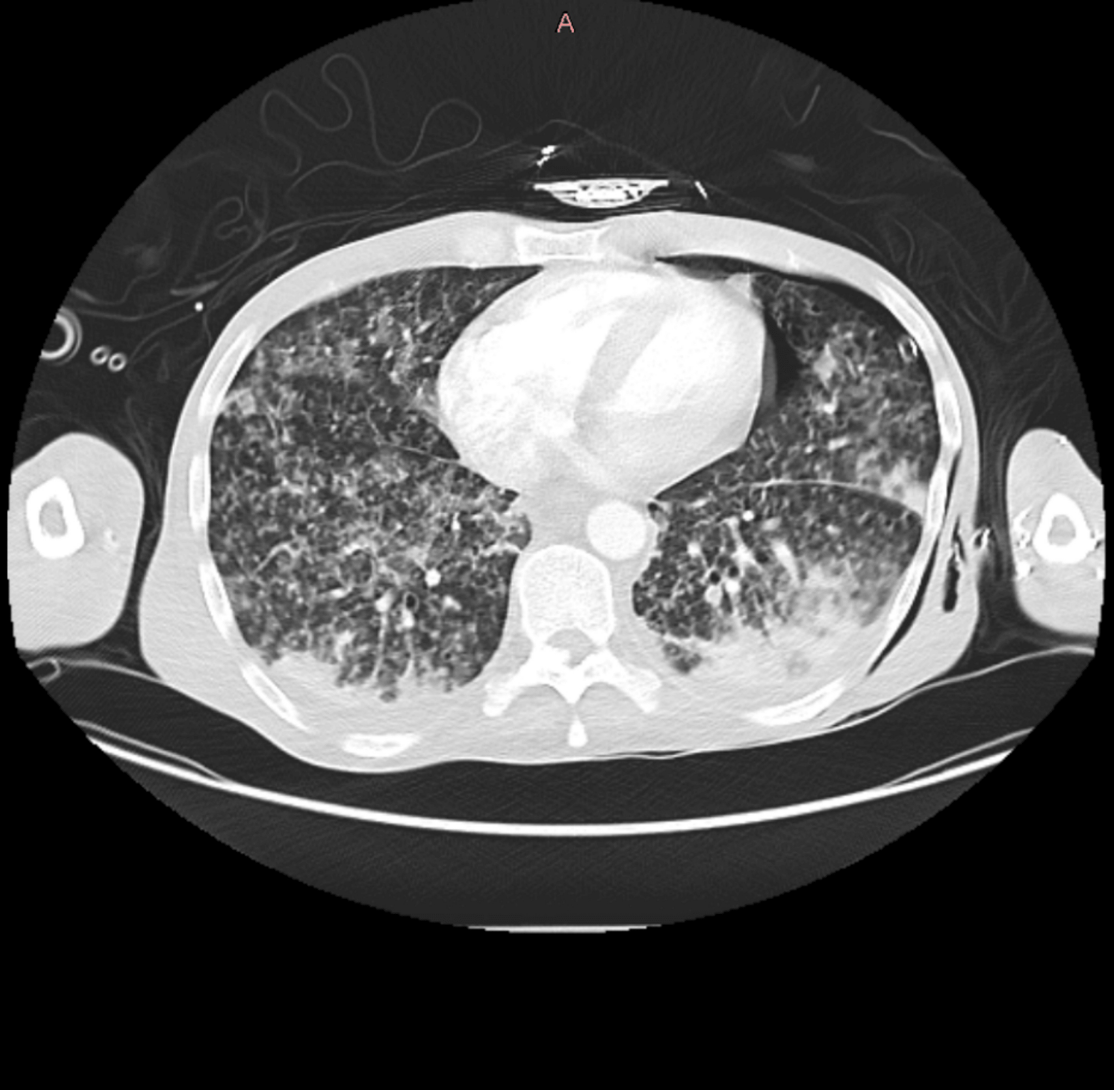
Repeat CT thorax done (08/03/2024) showing disease progression

A CT-TAP scan (Figure 4) revealed extensive bilateral pulmonary shadowing that appeared to have worsened compared to the previous scan, with areas of confluent consolidation suggesting progressive miliary TB. Mild bilateral basal effusions and mediastinal and hilar lymphadenopathy were noted, along with filling defects in the right lower lobe arteries indicating pulmonary embolism and a small left-sided pneumothorax. The left adrenal gland appeared bulky, and multiple cysts were observed in the mildly enlarged spleen. The small bowel was distended, with some non-dilated loops in the distal small bowel, suggesting small bowel obstruction with a zone of transition in the right lower abdomen. The liver appeared normal, with no signs of ascites.

Laboratory results revealed worsening liver function and persistently elevated CRP, indicating ongoing inflammation. Additionally, blood cultures were negative, and procalcitonin levels were mildly elevated (Table 2).

**Table 2:** Laboratory values of the second admission in comparison to the first admission

Test	First Presentation (18/01/2024)	Second Presentation (07/03/2024)	Reference Range	Abnormality (2nd Admission)
ALP	985 IU/L	1067 IU/L	30-130 IU/L	Above Normal
ALT	78 IU/L	109 IU/L	0-41 IU/L	Above Normal
GGT	338 IU/L	218 IU/L	0-71 IU/L	Above Normal
CRP	256 mg/L	143 mg/L	0-5 mg/L	Above Normal
WCC	7.7 × 10⁹/L	9.3 × 10⁹/L	4-10 × 10⁹/L	Normal
Hb	115 g/L	103 g/L	130-180 g/L	Below Normal
PLT	210 × 10⁹/L	234 × 10⁹/L	150-450 × 10⁹/L	Normal
Neutrophils	6.1 (81%)	8.6 (93%)	1.8-7.7 × 10⁹/L	Above Normal
Lymphocytes	1.1 (14%)	0.4 (4%)	1-4.8 × 10⁹/L	Below Normal

While, in the ward, the patient collapsed, experiencing oxygen desaturation to 71%. A repeat chest X-ray revealed a large left-sided pneumothorax, requiring urgent intervention from the intensive care team for bedside chest drain insertion. Following further deterioration and a decline in the Glasgow Coma Scale (GCS), the patient was intubated and transferred to the Intensive Care Unit (ICU). Microbiological consultation led to the initiation of rifampicin 600 mg once daily, levofloxacin 500 mg twice daily, isoniazid 300 mg once daily, and ethambutol 900 mg once daily to treat disseminated TB. Despite extensive medical interventions and ICU support, the patient's condition continued to deteriorate, resulting in multiorgan failure and ultimately leading to his death on March 15, 2024.

## Discussion

The pleiotropic cytokine TNF-α plays a significant role in the pathophysiology of AS, inflammatory bowel disease (IBD), rheumatoid arthritis (RA), and various other immune-mediated or inflammation-related conditions. Evidence suggests that individuals receiving TNF-α antagonists have an elevated risk of developing TB [5].

Prior to initiating anti-TNF-α treatment, all patients should adhere to the British Society for Rheumatology (BSR) guidelines. Tuberculin tests are generally not recommended for most rheumatology patients. Instead, the assessment of TB risk should be conducted through clinical history and examination, a chest X-ray, and, if necessary, the use of an algorithm from the guidelines that weighs the risk of chemoprophylaxis against the risk of TB. In any hospital setting, it is essential to consider the resource implications of increasing consultations with TB specialists [6]. However, it might be worth considering the use of interferon-gamma release assay (IGRA) tests or tuberculin tests in patients prior to initiating anti-TNF therapy in the future.

In this case, due to the patient's inadequate response to NSAIDs for managing AS, the decision was made to initiate anti-TNF-α therapy. This action aligns with the BSR guidelines, which required the patient to undergo pre-anti-TNF-α screening that included hepatitis screening and a chest X-ray. Given the patient's diagnosis of ankylosing spondylitis and sole reliance on nonsteroidal anti-inflammatory medications, along with the absence of recent TB exposure, travel history, or contact with TB cases, and considering the patient's White-British ethnicity, there was no justification for latent TB testing via IGRA test or Mantoux test. Therefore, the decision to initiate anti-TNF-α treatment was based on the patient's clinical history and the normal findings from the chest X-ray.

The UK is known for its cultural diversity, attracting individuals from countries where TB is endemic. Prior to their arrival to the UK, migrants who arrive via a formal visa route are required to undergo a chest X-ray to screen for TB. However, migrants arriving via unofficial ways are excluded from the pre-entry screening program; these individuals may originate from high-incidence countries and/or face intricate risk factors associated with their migration journey, hence elevating their susceptibility to TB [7]. In addition, nearly 80% of active TB cases reported in England occurred in individuals born outside the UK, where incidence rates remained elevated and stable [8]. Consequently, interactions with these individuals can pose a risk of TB transmission, even to those who have not traveled to endemic areas. This risk is especially significant for immunocompromised individuals, and medications such as adalimumab and infliximab significantly increase the risk of contracting TB [9]. Therefore, it is crucial to consider comprehensive screening for latent TB in these patients. Given their immunocompromised status and increased vulnerability to TB reactivation, there is a possibility that screening for latent TB could yield false-negative results [10]. This highlights the need to review and consider the initiation of TB chemoprophylaxis for these high-risk patients.

In this case, the patient's anti-TB medications were halted for five weeks due to the results of abnormal liver function, in accordance with hospital protocol. The discontinuation of all medications was deemed necessary following instances of hepatotoxicity, and the reintroduction of treatment was postponed until all biochemical markers returned to normal levels. It is widely acknowledged that the risk of adverse effects must be carefully balanced against the benefits of effective TB treatment. A prolonged interruption in treatment raises the risk of developing unintended drug resistance and may extend the overall duration of therapy. Therefore, when the anti-TB medications were resumed, second-line fluoroquinolones were added to the regimen to help reduce the risk of drug resistance [11].

## Conclusions

This case study underscores the critical need for comprehensive TB screening and monitoring in immunocompromised patients receiving TNF-α inhibitors, particularly adalimumab. The rapid progression of disseminated TB in this patient, despite prior screening, highlights the limitations of standard assessments such as chest X-rays and the necessity for thorough evaluations. Given the diverse population in the UK, immunocompromised individuals face an elevated risk of TB reactivation, regardless of exposure history, necessitating a proactive approach to TB screening and potential chemoprophylaxis.

Healthcare providers must adhere to the BSR guidelines, focusing on comprehensive clinical history and examination to assess TB risk effectively. Balancing the management of TB treatment adverse effects with the risk of drug resistance is essential for improving patient outcomes. Clinicians should maintain vigilance for TB symptoms in immunosuppressed patients and collaborate with TB specialists to optimize treatment strategies, reinforcing the importance of preventive measures in mitigating the risks associated with anti-TNF therapy.
